# Functional status of Indian children with juvenile idiopathic arthritis

**DOI:** 10.1515/rir-2024-0016

**Published:** 2024-07-15

**Authors:** Debadyuti Datta, Moksuda Khatun, Biswabandhu Bankura, Mihir Sarkar, Avijit Hazra, D Ivan M, Manab Nandy, Rakesh Mondal

**Affiliations:** Department of Paediatrics, Medical College Kolkata, Kolkata, India; Multidisciplinary Research Unit, Medical College Kolkata, Kolkata, India; Department of Pharmacology, IPGMER SSKM Hospital, Kolkata, India; Department of Biotechnology, Delhi Technological University, New Delhi, Delhi, 110042 India; Department of Pharmacology, Medical College Kolkata, Kolkata, India

**Keywords:** CHAQ disability index, juvenile idiopathic arthritis, functional status

## Abstract

**Background and Objectives:**

The functional disability status of Indian children with juvenile idiopathic arthritis is unidentified. In this cross-sectional study functional capacity of 60 juvenile idiopathic arthritis patients was assessed by the Childhood Health Assessment Questionnaire.

**Methods:**

A total of 60 juvenile idiopathic arthritis patients aged ranges from 1 to 12 years were recruited from a teaching hospital in eastern India. A childhood health assessment questionnaire was used to assess the functional health of children. Pain, patient’s/parent’s global assessment of general well-being, and physician’s global assessment were assessed.

**Results:**

Childhood health assessment questionnaire disability index for oligoarticular juvenile idiopathic arthritis differed significantly from polyarticular juvenile idiopathic arthritis (*P* < 0.001), systemic-onset juvenile idiopathic arthritis (*P* = 0.018) and undifferentiated juvenile idiopathic arthritis (*P* < 0.001). There was a good to a strong positive correlation between the childhood health assessment questionnaire disability index with pain score, patient’s/parent’s global assessment score, and physician global assessment score for the total juvenile idiopathic arthritis cohort. regarding juvenile idiopathic arthritis subtypes, significant correlations were noted between the childhood health assessment questionnaire disability index with the patient’s/parent’s global assessment and physician’s global assessment (except for enthesitis-related arthritis).

**Conclusions:**

Assessment and documentation of the functional health status of juvenile idiopathic arthritis patients will improve the management of the disease.

## Introduction

Juvenile idiopathic arthritis (JIA) is universal childhood morbidity. It is categorized into seven groups; each subtype is characterized by specific features, disabilities, and complications.^[1]^ Studies revealed that up to 70% of JIA patients continue to claim disability and restriction of their activities.^[2]^ High functional disability leads to poor academic performance and school absenteeism.^[3]^ The Childhood Health Assessment Questionnaire (CHAQ) is perhaps the most practiced questionnaire in clinical studies to measure functional outcomes in JIA.^[2,3]^ Study from India addressing functional disability in JIA patients is lacking. We addressed this lacuna.

## Methods

We conducted this cross-sectional study in a teaching hospital in eastern India from February 2022 to January 2023, over a year. JIA (International League of Associations for Rheumatology (ILAR) 2001 criteria) patients, 1 to 12 years of age, and their primary caregivers were included. The presence of cognitive disability and significant comorbidity were excluded. Functional health status was assessed by CHAQ which gives a disability index (DI). Pain, Patient’s/Parent’s Global Assessment (PtGA) of general well-being, and Physicians Global Assessment (PGA) were assessed. The study protocol was endorsed by the Institutional Ethics Committee for clinical research (MC/KoL/IEC/Non-Span/368/11–2016). Data were collected by a single investigator using a pretested structured interview schedule. To make the questionnaire user-friendly, CHAQ was translated (from English) into Bengali and Hindi languages with an adaption to Indian culture and lifestyle. Discrepancies in translation were resolved through consensus and the questionnaire was validated through pilot testing. Data have been summarized using descriptive statistics. Multiple JIA subgroups were compared by using the Kruskal-Wallis analysis of variance. Dunn’s test was used to pinpoint which pairwise differences were statistically significant. The strength of the association between numerical variables was quantified by Spearman’s rank correlation coefficient. In all comparisons, statistical significance was inferred if the p-value was < 0.05.

## Results

Out of 70 (10 excluded; non-availability of consent = 3, lack of defined caregiver = 4, presence of disabling comorbidity = 30), 60 children (53.5% males) and their caregivers (93.3% females, 86.7% mothers) were interviewed. The mean age of patients and caregivers was 8.5 and 35.9 years, respectively. JIA subtypes encountered were oligoarticular (28.3%), polyarticular (23.3%), systemic onset JIA (SoJIA; 23.3%) undifferentiated (16.7%), and enthesitis-related arthritis (ERA; 8.3%). Disease burden parameters of patients in different JIA subgroups are summarized in Table 1. CHAQ DI for oligoarticular JIA differed significantly from polyarticular JIA (*P* < 0.001), SoJIA (*P* = 0.018), and undifferentiated JIA (*P* < 0.001). There was a good to strong positive correlation of CHAQ DI with pain score, PtGA score, and PGA score for the total JIA cohort. Regarding JIA subtypes, significant correlations were noted between CHAQ DI and PtGA (except ERA) and PGA (except ERA and undifferentiated JIA). However, for individual subtypes, correlations between disability index and pain scores were weak.

**Table 1:** Summary of disease burden parameters for Juvenile Idiopathic Arthritis cases.

Parameter	Oligoarticular JIA (*n* = 17)	Polyarticular JIA (*n* = 14)	Systemic onset JIA (*n* = 14)	Undifferentiated JIA (n = 10)	*P*-value
CHAQ-DI					< 0.001
Range	0.80–2.00	1.80–2.50	1.70–2.20	0.60–2.70	
Mean ± SD	1.08 ± 0.28	2.24 ± 0.22	1.94 ± 0.18	2.11 ± 0.65	
Median (IQR)	1.00 (0.90–1.10)	2.20 (2.20–2.40)	1.95 (1.80–2.10)	2.30 (2.00–2.60)	
Pain Score					< 0.001
Range	1.50–6.00	5.00–7.50	5.00–7.10	3.00–8.00	
Mean ± SD	3.58 ± 1.20	6.56 ± 0.69	6.25 ± 0.62	5.88 ± 1.34	
Median (IQR)	3.60 (2.60–4.60)	6.65 (6.00–7.00)	6.35 (6.00–6.70)	5.95 (5.00–6.90)	
PtGA Score					< 0.001
Range	0.50–03.00	5.00–8.50	6.70–8.50	4.00–7.20	
Mean ± SD	1.82 ± 0.73	6.99 ± 0.87	7.60 ± 0.52	6.20 ± 0.88	
Median (IQR)	1.90 (1.05–2.55)	6.95 (6.70–7.70)	7.65 (7.10–8.00)	6.40 (5.90–6.80)	
PGA Score					< 0.001
Range	0.50–02.50	5.00–8.50	6.50–8.00	3.00–7.00	
Mean ± SD	1.73 ± 0.53	6.80 ± 1.01	7.37 ± 0.45	5.70 ± 1.20	
Median (IQR)	1.50 (1.50–2.00)	7.00 (5.70–7.50)	7.30 (7.00–7.70)	6.00 (5.50–6.70)	

IQR, Interquartile range; SD, Standard deviation; JIA, Juvenile idiopathic arthritis; CHAQ-DI, Childhood Health Assessment Questionnaire Disability Index; PtG , patient’s/ parent’s global assessment; PGA, physician global assessment. Pain Score, PtGA Score and PGA Score were all recorded on 10 cm visual analog scales. *P* value in the last column is from multiple comparison with Kruskal-Wallis one-way analysis of variance.

## Discussion

Polyarticular JIA imposed the highest functional disability in terms of CHAQ DI when various JIA subgroups were compared, whereas patients with oligoarticular JIA had the lowest disability. This observation is consistent with that of previous studies.^[3–6]^ A French study documented that ERA patients had the worst scores for both pain and well-being on the visual analogue scale (VAS) scale. Because of differences in JIA disease activity, subtype distribution, and assessment methodology, precise comparison with pre-existing studies is not always possible.^[4]^ In JIA subtypes, analysis significant correlations were noted between CHAQ DI and PtGA (except ERA) and PGA (except ERA and undifferentiated JIA). However, for individual subtypes, correlations between

DI and pain score were weak. This pattern broadly matches studies from other countries such as France,^[4]^ Canada,^[7]^ and Thailand.^[3]^ There are minor conflicts with previous studies regarding CHAQ DI correlation with other functional health parameters and physician-assessed disease activity (i.e., PGA) as the degree of correlation varies across studies.^[7]^ Contrary to this study, Boiu *et al*^[4]^ reported a good correlation between CHAQ DI and VAS pain score within each JIA category.

However, this study has its share of limitations. It was a single tertiary care center-based study with a limited sample size and children up to twelve years of age were included.

## Conclusion

In our study significant correlations were noted between CHAQ DI and PtGA (except ERA) and PGA (except ERA and undifferentiated JIA).

The work is a pioneering effort in documenting the functional health status of JIA patients in Indian scenarios. Assessment and documentation of the functional health status of juvenile idiopathic arthritis patients will improve the management of the disease.
